# Modeling Predictive Age-Dependent and Age-Independent Symptoms and Comorbidities of Patients Seeking Treatment for COVID-19: Model Development and Validation Study

**DOI:** 10.2196/25696

**Published:** 2021-03-25

**Authors:** Yingxiang Huang, Dina Radenkovic, Kevin Perez, Kari Nadeau, Eric Verdin, David Furman

**Affiliations:** 1 Buck Institute for Research on Aging Novato, CA United States; 2 Guy’s & St Thomas’ NHS Foundation Trust London United Kingdom; 3 King’s College London London United Kingdom; 4 Division of Pulmonary, Allergy and Critical Care Medicine Sean N Parker Center for Allergy and Asthma Research Stanford University Stanford, CA United States

**Keywords:** clinical informatics, predictive modeling, COVID-19, app, model, prediction, symptom, informatics, age, morbidity, hospital

## Abstract

**Background:**

The COVID-19 pandemic continues to ravage and burden hospitals around the world. The epidemic started in Wuhan, China, and was subsequently recognized by the World Health Organization as an international public health emergency and declared a pandemic in March 2020. Since then, the disruptions caused by the COVID-19 pandemic have had an unparalleled effect on all aspects of life.

**Objective:**

With increasing total hospitalization and intensive care unit admissions, a better understanding of features related to patients with COVID-19 could help health care workers stratify patients based on the risk of developing a more severe case of COVID-19. Using predictive models, we strive to select the features that are most associated with more severe cases of COVID-19.

**Methods:**

Over 3 million participants reported their potential symptoms of COVID-19, along with their comorbidities and demographic information, on a smartphone-based app. Using data from the >10,000 individuals who indicated that they had tested positive for COVID-19 in the United Kingdom, we leveraged the Elastic Net regularized binary classifier to derive the predictors that are most correlated with users having a severe enough case of COVID-19 to seek treatment in a hospital setting. We then analyzed such features in relation to age and other demographics and their longitudinal trend.

**Results:**

The most predictive features found include fever, use of immunosuppressant medication, use of a mobility aid, shortness of breath, and severe fatigue. Such features are age-related, and some are disproportionally high in minority populations.

**Conclusions:**

Predictors selected from the predictive models can be used to stratify patients into groups based on how much medical attention they are expected to require. This could help health care workers devote valuable resources to prevent the escalation of the disease in vulnerable populations.

## Introduction

The COVID-19 pandemic, caused by SARS-CoV-2, continues to burden medical institutions around the world by increasing total hospitalization and intensive care unit (ICU) admissions [1-9]. A better understanding of symptoms, comorbidities, and medications used for pre-existing conditions of patients with COVID-19 could help health care workers identify patients at increased risk of developing a more severe case of the disease [10,11]. Here, we have used self-reported data (symptoms, medications, and comorbidities) from 11,194 users of the COVID-19 Symptom Tracker app [12] to identify previously reported and novel features predictive of patients being admitted to a hospital. Despite the previously reported association between age and more severe disease phenotypes [13-18], we found that a patient’s age, sex, and ethnic group were minimally predictive when compared to the patient’s symptoms and comorbidities. The most important variables selected by our predictive models were fever, the use of immunosuppressant medication, the use of a mobility aid, shortness of breath, and fatigue. It is anticipated that early administration of preventative treatment in patients with COVID-19 who exhibit a high risk of a severe disease signature may prevent disease progression. Therefore, the variables found could provide clinical decision support.

## Methods

### Cohort

The COVID-19 Symptom Tracker is a smartphone app to which individuals from the United Kingdom and United States can submit their symptoms daily [19-22]. A total of 3,643,842 users had signed up for the app as of November 1, 2020 [12]. A user can make entries in the app and record features such as symptoms, comorbidities, medications taken for pre-existing conditions, and demographics. Users can log entries whether they have tested positive for COVID-19 or not. They can also create multiple entries per day or one entry every few days. Data from March 24, 2020, to November 1, 2020, were accessed from The Health Data Research Hub for Respiratory Health, in partnership with Secure Anonymised Information Linkage (SAIL) Databank. There were users from both the United States and the United Kingdom, but we only used the UK population to maintain the homogeneity of the data set (Figure S1 in Multimedia Appendix 1). The US population was small and could have a different disease expression than that caused by the virus strain in the United Kingdom. Therefore, we excluded the small US population.

For the study cohort, we extracted all users who tested positive for COVID-19 (n=11,194). Of those users who were positive for COVID-19, some cases were severe enough to require seeking treatment at a hospital (hereinafter referred to as seeking treatment) while others managed their disease at home (Figure S1 in Multimedia Appendix 1). The features we used in all subsequent models are listed in Tables 1-4. All features were binary except for age and BMI, which were continuous, and shortness of breath, fatigue, race, and gender, which were categorical.

**Table 1:** Features of symptoms investigated in relation to whether a user was seeking treatment. All features were binary except for shortness of breath and fatigue, which were categorical.

Symptoms			Managed at home (N=10,636), n (%)		Admitted into hospital setting (N=558), n (%)
**Fever**					
	1	2761 (26.4)		155 (29.0)	
	N/A^a^	42 (0.40)		80 (15.0)	
**Persistent cough**					
	1	5429 (52.1)		255 (47.7)	
	N/A	0 (0.0)		80 (15.0)	
**Diarrhea**					
	1	2944 (28.3)		178 (33.3)	
	N/A	0 (0.0)		80 (15.0)	
**Delirium**					
	1	1888 (18.1)		137 (25.6)	
	N/A	0 (0.0)		80 (15.0)	
**Skipped meals**					
	1	3967 (38.1)		218 (40.7)	
	N/A	0 (0.0)		80 (15.0)	
**Abdominal pain**					
	1	2452 (23.5)		129 (24.1)	
	N/A	116 (1.11)		83 (15.5)	
**Chest pain**					
	1	2452 (23.5)		129 (24.1)	
	N/A	116 (1.1)		83 (15.5)	
**Loss of smell**					
	1	6251 (60.0)		217 (40.6)	
	N/A	95 (0.9)		83 (15.5)	
**Headache**					
	1	4326 (41.5)		250 (43.0)	
	N/A	95 (0.9)		83 (15.5)	
**Sore throat**					
	1	3862 (37.1)		158 (29.5)	
	N/A	344 (3.30)		94 (17.6)	
**Unusual muscle pains**					
	1	3442 (33.1)		168 (31.4)	
	N/A	1215 (11.7)		116 (21.7)	
**Shortness of breath**					
	N/A	0 (0.0)		80 (15.0)	
	No	6771 (65.0)		219 (40.9)	
	Mild	2945 (28.3)		156 (29.2)	
	Significant	626 (6.01)		65 (12.1)	
	Severe	71 (0.7)		15 (2.8)	
**Fatigue**					
	N/A	0 (0.0)		80 (15.0)	
	No	5922 (56.9)		210 (39.3)	
	Mild	3712 (35.6)		178 (33.3)	
	Severe	779 (7.5)		67 (12.5)	

^a^N/A: data not available or missing.

**Table 2:** Features of comorbidities investigated in relation to whether a user was seeking treatment. All features were binary.

Comorbidities		Managed at home (N=10,636), n (%)	Admitted into hospital setting (N=558), n (%)
**Has diabetes**			
	1	370 (3.6)	48 (9.0)
	N/A^a^	26 (0.2)	1 (0.2)
**Has heart disease**			
	1	209 (2.0)	40 (7.5)
	N/A	26 (0.2)	1 (0.2)
**Has lung disease**			
	1	1446 (13.9)	122 (22.8)
	N/A	26 (0.2)	1 (0.2)
**Is a smoker**			
	1	294 (2.8)	21 (3.9)
	NA	5241 (50.3)	265 (49.5)
**Undergoing chemotherapy**			
	1	29 (0.3)	12 (2.2)
	N/A	5184 (49.8)	244 (45.6)
**Has kidney disease**			
	1	91 (0.9)	19 (3.6)
	N/A	26 (0.2)	1 (0.2)
**Housebound**			
	1	538 (5.2)	102 (19.1)
	N/A	19 (0.2)	0 (0.0)
**Uses a mobility aid**			
	1	183 (1.8)	77 (14.4)
	N/A	19 (0.2)	0 (0.0)
**Limited activity**			
	1	886 (8.5)	137 (25.6)
	N/A	26 (0.2)	1 (0.2)

^a^N/A: data not available or missing.

**Table 3:** Features of demographics investigated in relation to whether a user was seeking treatment. Age and BMI were continuous features, while race and gender were categorical.

Demographics		Managed at home (N=10,636)	Admitted into hospital setting (N=558)
**Gender, n (%)**			
	Female	2981 (28.4)	219 (40.9)
	Male	7455 (71.6)	316 (59.1)
**Race, n (%)**			
	UK Asian	519 (5.0)	29 (5.4)
	UK Black	116 (1.1)	9 (1.7)
	UK mixed, White/Black	56 (0.5)	1 (0.2)
	UK mixed, other	116 (1.1)	4 (0.7)
	UK White	9346 (89.7)	472 (88.2)
	UK Chinese	43 (0.4)	4 (0.7)
	UK Middle Eastern	89 (0.8)	8 (1.5)
	Other	97 (0.9)	5 (0.9)
	Prefer not to say	33 (0.3)	3 (0.6)
Age (years), mean (SD)		40.2 (13.6)	47.8 (18.8)
BMI, mean (SD)		26.3 (6.9)	27.9 (8.2)

**Table 4:** Features of medication history investigated in relation to whether a user was seeking treatment. All features were binary.

Medication		Managed at home (N=10,636), n (%)	Admitted into hospital setting (N=558), n (%)
**Takes corticosteroids**			
	1	719 (6.9)	62 (11.6)
	N/A^a^	26 (0.2)	1 (0.2)
**Takes immunosuppressants**			
	1	287 (2.8)	65 (12.1)
	N/A	26 (0.2)	1 (0.2)
**Takes any blood pressure medications**			
	1	721 (6.9)	93 (17.4)
	N/A	1022 (9.8)	54 (10.1)

^a^N/A: data not available or missing.

### Data Processing

We used comorbidities, demographics, and symptoms to predict whether a user would seek treatment using predictive models. For data preprocessing, we first divided the patients with COVID-19 into two groups: (A) negative for seeking treatment, including patients who were positive for COVID-19 who were strictly at home, without ever having to be admitted to a hospital setting (n=10,636) and (B) positive for seeking treatment, including users who were positive for COVID-19 who reported being in a hospital setting (n=558). The average age of group A was 40.2 (SD 13.6) years compared to 47.8 (SD 18.8) years for group B. For group A, we used comorbidities, demographics, and symptoms recorded in the patient's last entry, and for group B, we used the same features as recorded in the patient’s last entry prior to the entry where the patient indicates seeking treatment (scenario 1). This means that for users who sought treatment, we used the time point right before a user indicated seeking treatment in a hospital setting and the features at that time point were used for analysis. For users who were always at home, we used the last time point and the features at that time point for analysis (Figure S2, top panel, in Multimedia Appendix 1). In what we call scenario 2, for users who were seeking treatment, if a user indicated that he/she had a feature in any of his/her entries before the day of seeking treatment, we labeled that feature as positive for that user. For users who were always at home, if he/she had a given feature in any entry, we labeled that feature as positive for that user (Figure S2, bottom panel, in Multimedia Appendix 1). Such processing only applied to symptoms since they can change daily, but not to comorbidities, pre-existing medication use, or demographic information.

### Imputations

Patients often neglect to report all available fields, so we used the multiple imputations method to impute missing values, a standard procedure to predict missing data using all other features (besides the outcome) that are not missing [23-25]. Multiple imputations were used to impute missing values. Instead of imputing the missing value with a single value, multiple imputations repeatedly sample the data *n* times and impute the missing values *n* times. Different types of data require different imputation methods. We used predictive mean matching for continuous numerical data (age, BMI), polytomous regression for unordered categorical data (gender, race), proportional odds model for ordered categorical data (fatigue, shortness of breath), and logistic regression for binary data (all other features). The predictive variables for the different methods would be all other independent variables, while the outcome would be the missing variable. The most stringent process would only impute the training set, but there were not enough complete instances to have both positive and negative cases; therefore, we imputed the training and testing sets together. Multiple imputations produce *n* imputations, and we pooled *n* imputed matrices together to form a larger training set. Some variables had a large percentage of missing values, as seen in Figure S3, bottom panel (Multimedia Appendix 1). Such variables produced wide ranges of distribution from one imputation to another that are too different from the original distribution (Figure S3, top panel, in Multimedia Appendix 1). Therefore, those variables were removed from the data sets. The final set of variables are all present in Tables 1-4.

### Imbalanced Classification

Class imbalance was a problem in our data set, where the minority class (individuals who sought treatment) constituted about 5% of the total COVID-19 population. Upon examining the distribution modality of our data set and discovering that the minority class is multimodal, we decided to use sampling methods to balance the data set and a binary classifier in the next section [26]. The sampling method we employed randomly undersamples the majority class and oversamples the minority class until the data is balanced and the total number of instances is equal to the original number of instances. Such a sampling method was applied in the following predictive model.

### Predictive Modeling

We performed an Elastic Net regularized binary classification to select for the most important features in predicting users who needed to seek medical treatment. The data set was divided into 10-fold cross-validation with 10 training and test sets (with a ratio of 70:30). In each of the cross-validations, the resampling method was applied to the training set. Parameters were then tuned for the Elastic Net classifier using the training set, producing the best predictive performance and the most parsimonious number of features. Two parameters can be tuned in Elastic Net, alpha (*α*) and lambda (*λ*). Alpha is the mixing parameter, indicating how much least absolute shrinkage and selection operator (LASSO) and Ridge regularization should contribute to the model, while lambda is the amount of shrinkage or regularization the model should apply as a whole. A series of alpha is used in each cross-validation in a grid search with values ranging from 0.0 to 1.0 in steps of 0.05. The alpha that produces the highest area under the receiver operating characteristic curve (AUROC) at the minimum lambda is chosen. For scenario 1, the best alpha is 0.1. Two common lambdas are generally used: the lambda that gives the best performance (lambda.min) or the lambda with the fewest features selected that is within one standard error of the best-performing lambda (lambda.1se). We used lambda.1se, the most generalizable model, to avoid overfitting and selecting the most salient variables. From each of the cross-validations, a slightly different set of features were selected even though the features with the highest coefficients were relatively consistent. To account for the slight inconsistencies, the final set of features consists of features that were selected in all 10 cross-validations. The final set of features were used to predict estimated probabilities in the 10 test sets and calculate subsequent performance metrics.

### Software

Analyses were all carried out in R (version 4.0.3). Packages used are listed as follows. For imputation, we used MICE (Multiple Imputation by Chained Equations; version 3.13.0). Imbalanced data set sampling was performed with ROSE (Random Over-Sampling Examples; version 0.0-3). For Elastic Net, we used glmnet (Lasso and Elastic-Net Regularized Generalized Linear Models; version 4.1).

## Results

### Elastic Net Results

**Figure 1:**
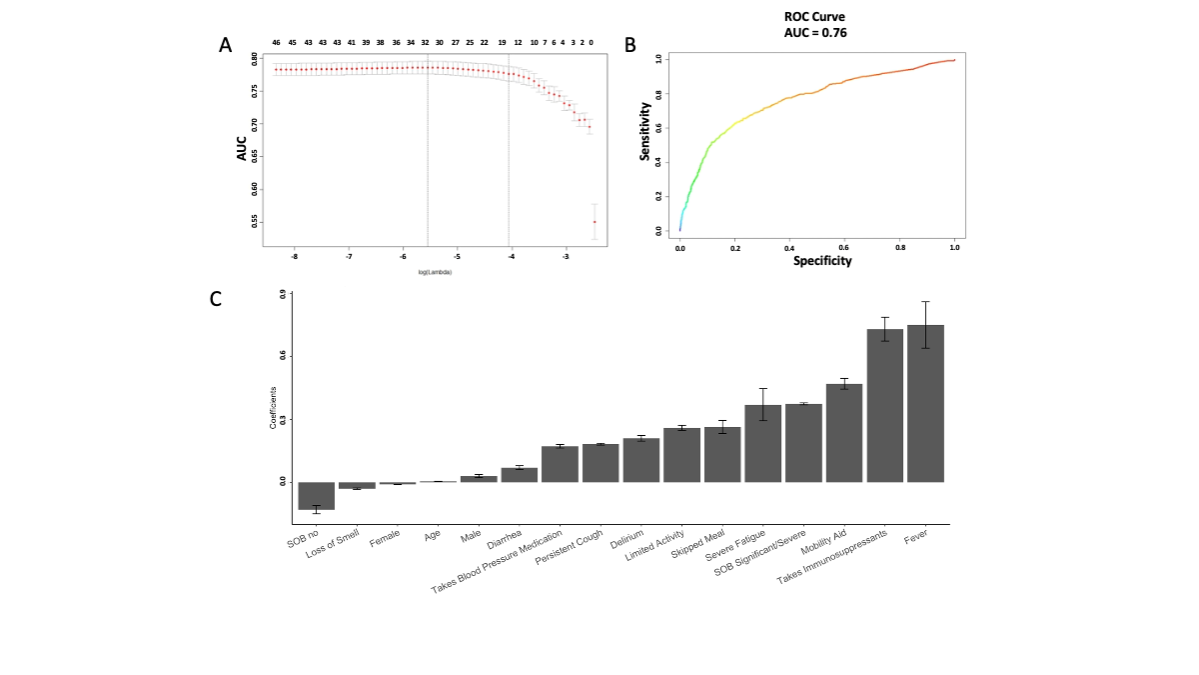
Elastic Net regression predictive performance and selected variables. We used Elastic Net regression where the outcome of seeking treatment or not was regressed on features in Tables 1-4. An example performance in terms of cross-validation AUC for Elastic Net classification on the training set across different values of lambda is shown in the top left. The final set of features selected from the Elastic Net model was applied on a holdout test data set and the resulting AUC is shown in the top right. The most important features selected by the Elastic Net model are shown at the bottom. Negative coefficients indicate a negative association with outcome and vice versa. AUC: area under the receiver operating characteristic curve; ROC: receiver operating characteristic; SOB: shortness of breath.

We were able to predict users seeking treatment with relatively good accuracy. The average cross-validated area under the receiver operating characteristic curve (cvAUC) for the training set at the optimal parameters was 0.752 (SD 0.021). An example of the lambda path from one Elastic Net training is shown in Figure 1A, with the most parsimonious model selecting 16 features. Using the final set of features selected (Figure 1C) to calculate estimated probabilities and the area under the curve (AUC) on the 10 test sets, a similar accuracy was obtained, with an average AUC of 0.745 (SD 0.033). An example of AUC on the test set is shown in Figure 1B. The most important variables of this signature as selected by our predictive algorithm were fever, the use of immunosuppressant medication, the use of a mobility aid, shortness of breath, and severe fatigue. Age had a relatively small regression coefficient, indicating that pre-existing clinical conditions and symptom presentation are much stronger predictors of seeking treatment. Unexpectedly, BMI was not selected as a significant predictor. Finally, female gender was negatively associated with seeking treatment. Elastic Net regression was also applied to scenario 2. The prediction performance is comparable to scenario 1, and the selected features were also very similar (Figure S6 in Multimedia Appendix 1).

### Logistic Regression Results

We next estimated the odds ratio from logistic regression using scenario 1 (Figure S4 in Multimedia Appendix 1). The most important features are consistent with the Elastic Net results. The modeling from logistic regression and Elastic Net regression using scenario 1 and 2 all selected similar features that are predictive of the outcome, lending robustness to the results.

### Longitudinal Analysis

**Figure 2:**
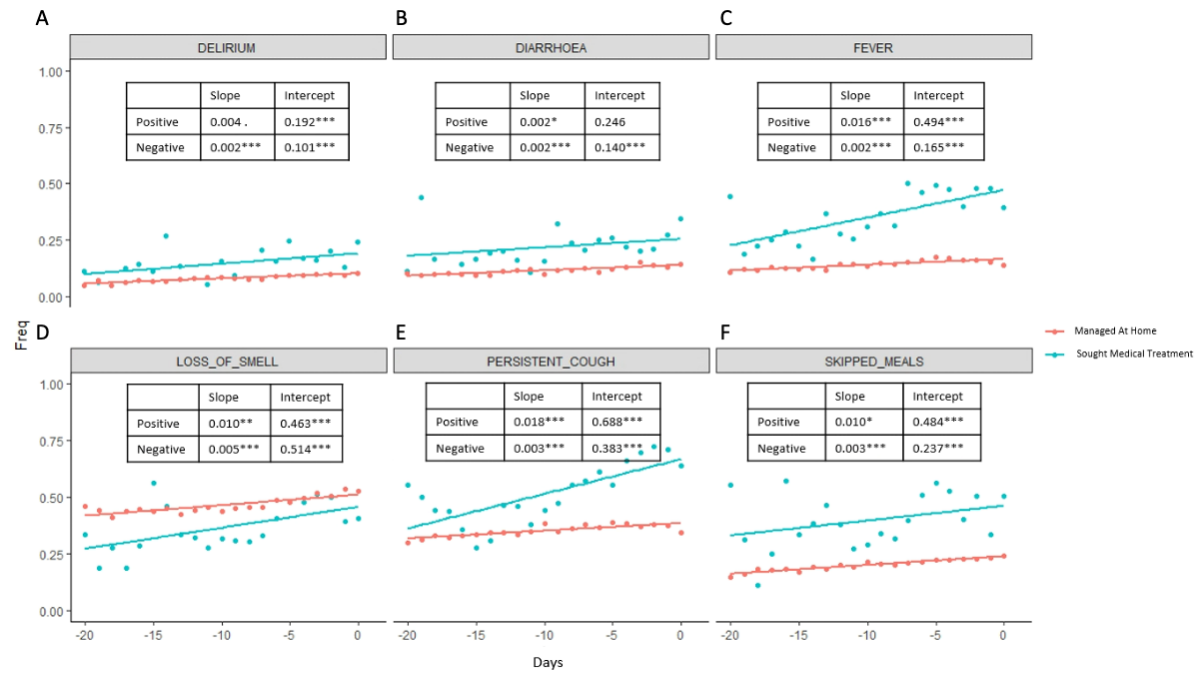
We analyzed all Elastic Net regression–selected symptoms for a 20-day window in a longitudinal manner, where day 0 for the positive group corresponds to the day when the user seeks treatment, and day 0 for the negative group corresponds to the last entry. The frequency of users having each feature for each day was plotted. A linear regression line where the frequency is regressed onto the days is plotted. The slopes and intercepts are labeled. Binary features of both positive and negative groups are plotted.

**Figure 3:**
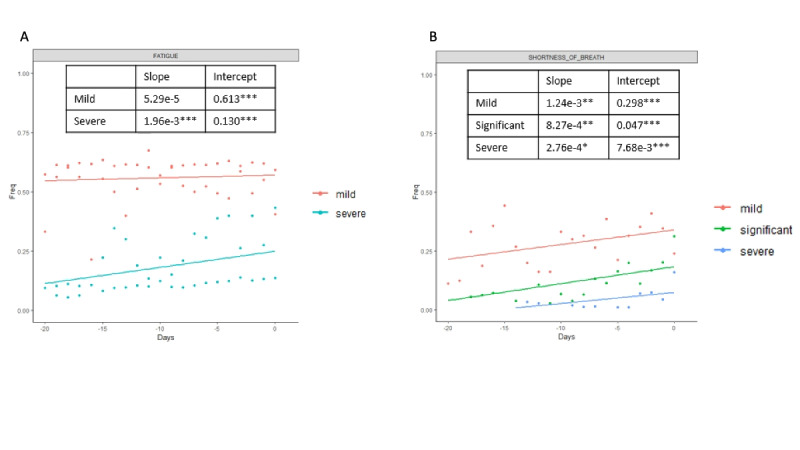
Categorical features of fatigue and shortness of breath are plotted for the positive group in longitudinal analysis.

**Figure 4:**
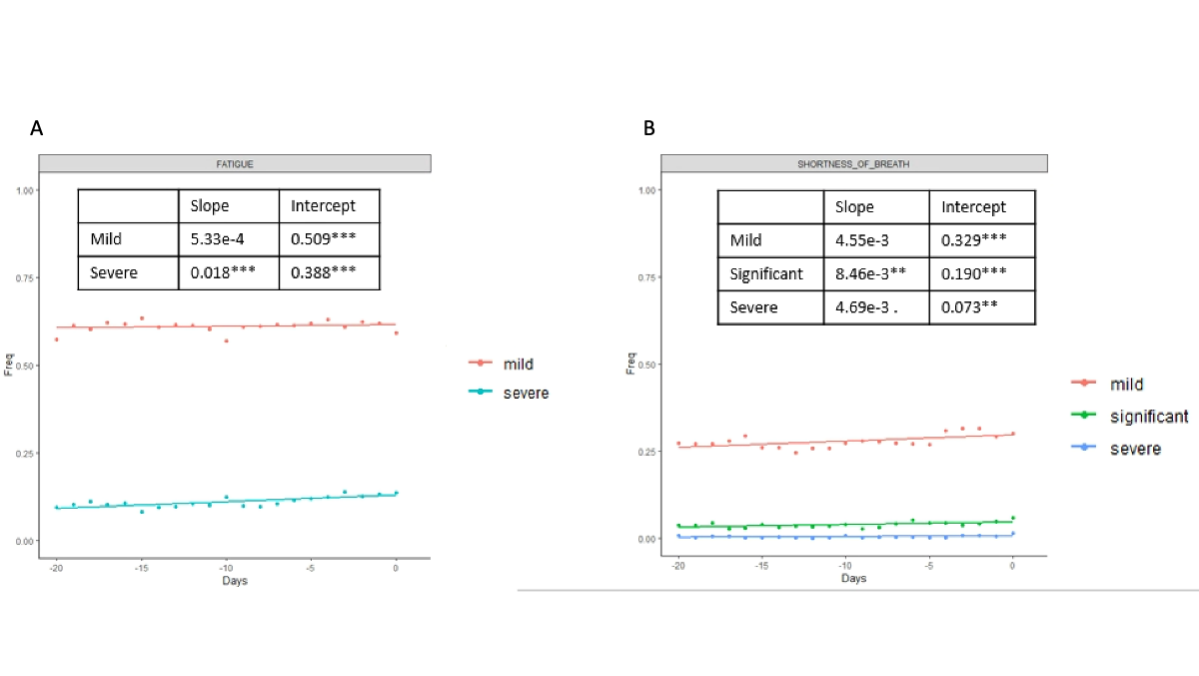
Categorical features of fatigue and shortness of breath are plotted for the negative group in longitudinal analysis.

To better understand the fluctuations in the symptoms selected by the Elastic Net model, we then analyzed the symptoms in a longitudinal manner. We examined a window of time beginning 20 days before the patient goes to a hospital setting (for positive cases), and 20 days before the last entry (for negative cases); the results are shown in Figures 2-4. For each day, we estimated the frequency of each symptom for the positive and negative groups. Day 0 for the positive group corresponds to the day when the user seeks treatment, and day 0 for the negative group corresponds to the last entry. Figure 2 shows positive and negative groups of binary variables. Figure 3 shows categorical variables of fatigue and shortness of breath for the positive group, and Figure 4 shows fatigue and shortness of breath for the negative group. A linear regression line is superimposed for each group where the frequency is regressed on the days.

Slope and intercepts are shown for comparison and their significance is evaluated using the likelihood ratio test (Figure S7 in Multimedia Appendix 1). The likelihood ratio test was used to compare whether there are statistically significant differences between the slopes of positive and negative cases in the trajectory analysis. Linear regression was used to quantify the association between days and frequency of each selected symptom in positive and negative cases. The likelihood ratio test was used to compare the linear regression model, where only the frequency of the feature was the independent variable, and the linear regression model, where the frequency of the feature and whether cases are positive or negative were the independent variables. The null hypothesis is that a linear model with only frequency of the feature as the independent variable is the superior model. The alternative hypothesis is that the superior model is the model with frequency of features and whether cases are positive or negative as independent variables. Rejection of the null hypothesis suggests that knowing the positive or negative cases predicts better frequency of the feature; therefore, the positive and negative cases are statistically different.

All differences between the two groups were significant except for mild fatigue. The slopes of the positive group were steeper than in the negative group in all the symptoms except for diarrhea, which indicates that the positive group had an increased frequency of symptoms that are indicative of severe COVID-19 cases as the disease progressed, while the frequency of the symptoms for the negative group stayed relatively stable. Not surprisingly, all the intercepts for the positive group are higher than for the negative group except for mild fatigue, further indicating that there are higher frequencies of COVID-19–related symptoms in users who were seeking treatment.

### Age-Related Variables

To understand the age effects better, given that age has little significance in predicting the outcome, we analyzed the association between age and the other features selected. We conducted an experiment where we divided all the users who were positive for COVID-19 into three age groups: young, middle-aged, and older adults (aged <30 years, 30< aged <50 years, and aged >50 years, respectively). Running univariate logistic regression where the outcome of seeking treatment is regressed onto each feature selected by the Elastic Net model shows that the coefficients of the features do not vary substantially between age groups (Figure S5 in Multimedia Appendix 1). Such results suggest that the features’ association with the outcome is not dependent on age.

**Figure 5:**
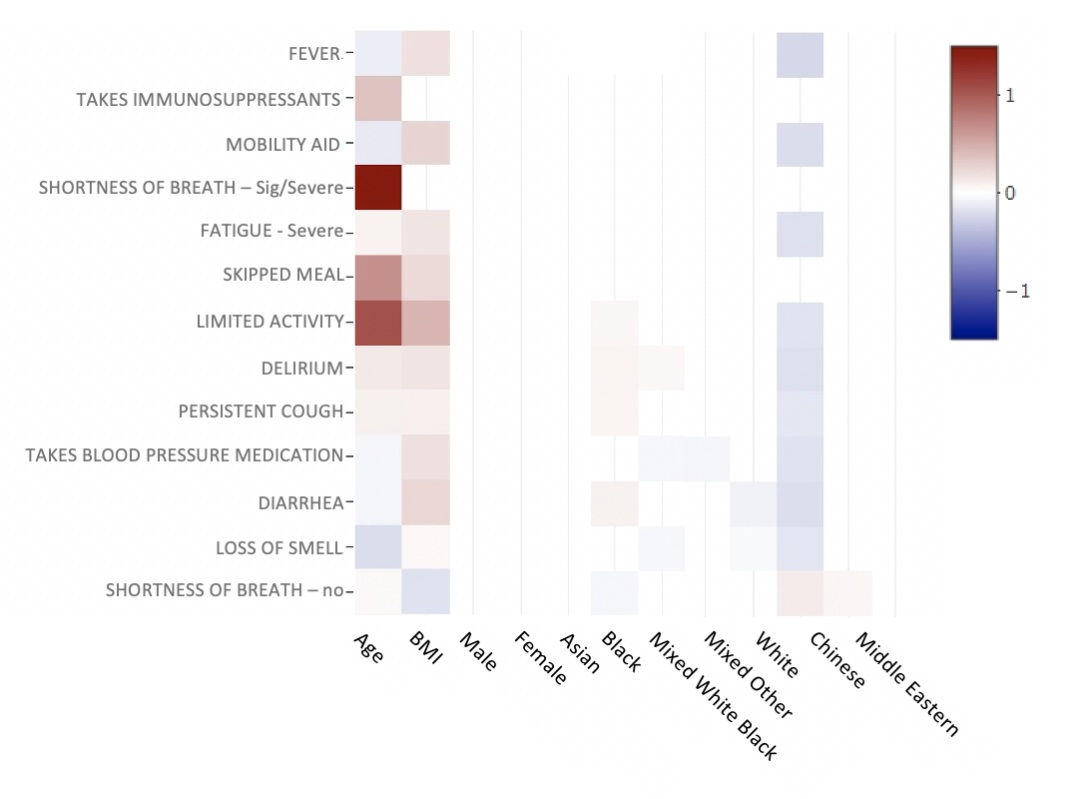
Multivariate logistic regression where each Elastic Net regression–selected feature is regressed onto demographic information such as age, BMI, gender, and race. Coefficients are plotted in a heat map. Only statistically significant associations are plotted. Age has a significant but weak association with many selected features. Users who identified as Black in the United Kingdom have many positive associations with high coefficients. Sig: significant.

SARS-CoV-2 has been shown to cause more severe disease in older adults [27]. Even though age was not a major contributor to the prediction of COVID-19–related treatment seeking, we explored whether age was associated with other features selected by the model. In conjunction, we also examined other demographic variables, such as race, BMI, and gender. We conducted multivariate logistic regression models where each of the features selected by the Elastic Net model was regressed on the demographic variables analyzed (Figure 5). Age was associated with 10/13 of the predictive features (*P*<.01). The most age-correlated features were use of a mobility aid, limited activity, blood pressure medication use, and immunosuppressant medication use. This indicates that age-related phenotypes in this cohort are associated with seeking treatment due to COVID-19. This emphasizes the fact that despite age, any population that expresses the features selected from our model could be susceptible to a more severe form of COVID-19. Understanding vulnerable young populations that are biologically older than their chronological age and exhibit features that are generally associated with the older population could help identify susceptible young populations.

In addition to age, being of Black ethnicity was associated with a number of features selected by the Elastic Net, such as a high frequency of delirium, limited activity, and blood pressure medication use. However, whether this is associated with socioeconomic status or an innate biological difference in people of African descent needs further investigation. The gender feature was a predictor of seeking treatment (Figure 1) but was not significantly correlated with any of the predictive features, suggesting that the sex of an individual affects other aspects of disease severity not evaluated in this study.

## Discussion

In this paper, we used Elastic Net classifier to identify features that are pertinent to users seeking medical treatment for COVID-19. A relatively small effect of the loss of smell feature associated with mild disease outcomes was found (Figure 1). Such findings have also been reported in recent studies [28,29]. Female gender also had a negative correlation with seeking treatment, consistent with recent findings in large populations [30,31]. In our data, gender did not correlate strongly with any features, indicating that there may be factors other than comorbidities or symptoms that enable females to have a better prognosis. Underlying immunological differences in females [32-35] could lead to the mounting of a better immune response and neutralization of the virus more efficiently than in men.

From our analyses, we have found features that are predictive of people having a severe enough case of COVID-19 to be admitted into a hospital setting. However, there are some features where additional research would help elucidate the mechanism behind their correlation. For example, immunosuppressant use was a major predictor of a patient seeking treatment; however, from the data, we cannot investigate whether patients taking these drugs are more prone to severe COVID-19 because of underlying autoimmune or autoinflammatory disease or because of the direct effect of the drug on the suppression of the inflammatory response. If the latter was true, immunosuppressant use might be ameliorating severe disease phenotypes that are frequently caused by cytokine storms [36], which could be attenuated by the use of immunosuppressant medication.

Besides the need for additional research into the mechanism behind some of the features associated with a more severe disease state, time is an important variable that is not explored in depth in this paper. A Cox survival analysis would be informative; however, the start time of each user is inconsistent, making the application of Cox survival analysis inappropriate. Some users’ first entries already indicate testing positive for COVID-19, with symptoms suggesting that they are already in the middle of the disease course, while others slowly develop symptoms and test positive for COVID-19 later in time.

There are also limitations to a mobile app and using whether users seek treatment as a proxy to accurately capture “severity.” Those who are able to manage their disease at home are considered less severe than those who sought treatment in a clinical or hospital setting. Such a proxy is different from the more formal severity scale developed by the World Health Organization. It is also conceivable that the sickest may not log their symptoms. However, we believe that self-reported data can provide more insight into when individuals with COVID-19 are sick enough to seek medical attention and what features are related to the change from self-care to medical treatment. Such higher resolution insight can provide those who have COVID-19 with warning signs of progressively worsening disease.

Age has been shown to be important in the severity of COVID-19 [13]. In our results, age shows a slight positive correlation with users seeking treatment. The difference between the average age of those who sought treatment and those who did not was relatively small, consistent with age not being a strong predictor. It is possible that the older population was less likely to use a smartphone app, leading to underrepresentation of the sick older population in our data set. To explore such a possibility, we accessed publicly available data on the age distribution of those infected with COVID-19 in the United Kingdom from March-September 2020 [37]. There is a bimodal distribution in the general UK population, with peaks at 50 and 80 years (average age of 57.6 years), while we only see a unimodal distribution in our data set, with the peak at 50 years (average age of 44.3 years; Figure S8 in Multimedia Appendix 1). A chi-square test comparing the COVID-19–positive population aged >60 years in the Tracker App versus the COVID-19–positive population aged >60 years in the UK general population also showed that there is a statistically significant difference (*P*<.001). This could explain the discrepancies between the small coefficient of importance for age in our model compared to previous literature, which were mostly published before September 2020, where age is a major factor in disease severity. Furthermore, the fact that age-associated variables outperform age in the prediction of a patient seeking treatment could also indicate that biological age or immunological age [38,39] may be appropriate measures for assessing an individual's prognosis. Chronological age is not a good representation of the condition of an individual. Biological age has been shown to be associated with all-cause mortality and susceptibility to diseases and may therefore be a more appropriate measure of how healthy an individual may be and how he/she might respond to COVID-19.

In conclusion, we identify age-dependent and age-independent sets of symptoms and comorbidities predictive of patients with COVID-19 seeking treatment. Our analyses show features that predict disease severity in advance, which could be used to predict severe cases of COVID-19 even in younger individuals who may not be labeled as high risk. A continued rise in the number of cases, as many societies struggle to balance reopening the economy and “flattening the curve,” places an enormous burden on health care systems around the world. Knowing the signs of possible severe cases, including the ones derived in this study, could help health care systems devote resources to intervening in potentially severe cases before they become costly to manage.

## Appendix

Multimedia Appendix 1 Supplementary materials.

## Abbreviations

AUC: area under the curve
AUROC: area under the receiver operating characteristic curve
cvAUC: cross-validated area under the receiver operating characteristic curve
ICU: intensive care unit
WHO: World Health Organization
