# Supplementary material for: Modeling Predictive Age-Dependent and Age-Independent Symptoms and Comorbidities of Patients Seeking Treatment for COVID-19: Model Development and Validation Study
Source: J Med Internet Res. 2021 Mar 25;23(3):e25696. doi: 10.2196/25696 (PMC7996196; doi:10.2196/25696)
Supplement: Multimedia Appendix 1 [file jmir_v23i3e25696_app1.docx]

**Multimedia Appendix 1**

**
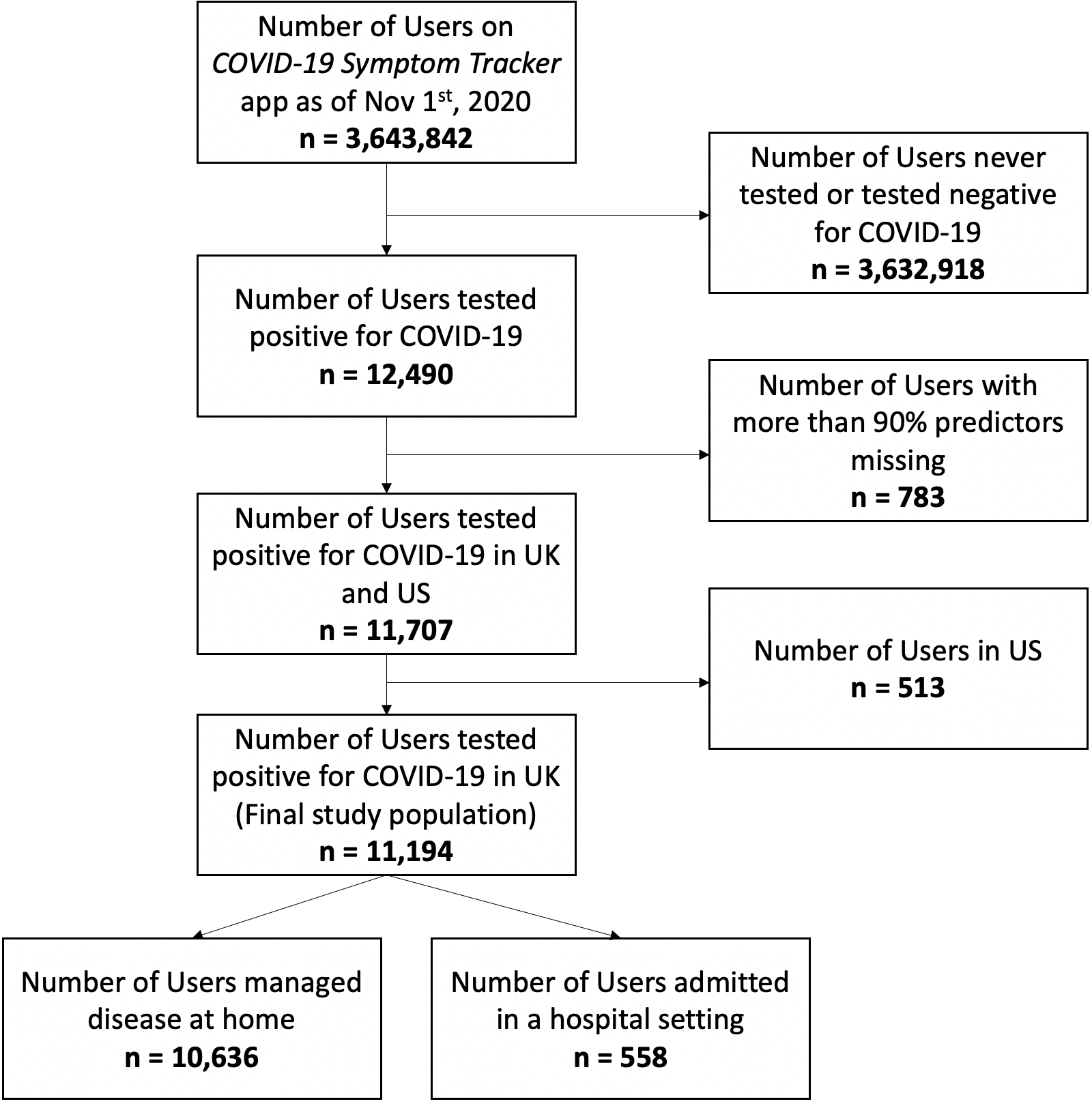
**

**Figure S1. Diagram of cohort with inclusion and exclusion criteria.** Only Users tested positive for COVID-19 were included. Users with too many predictors missing were excluded.

**
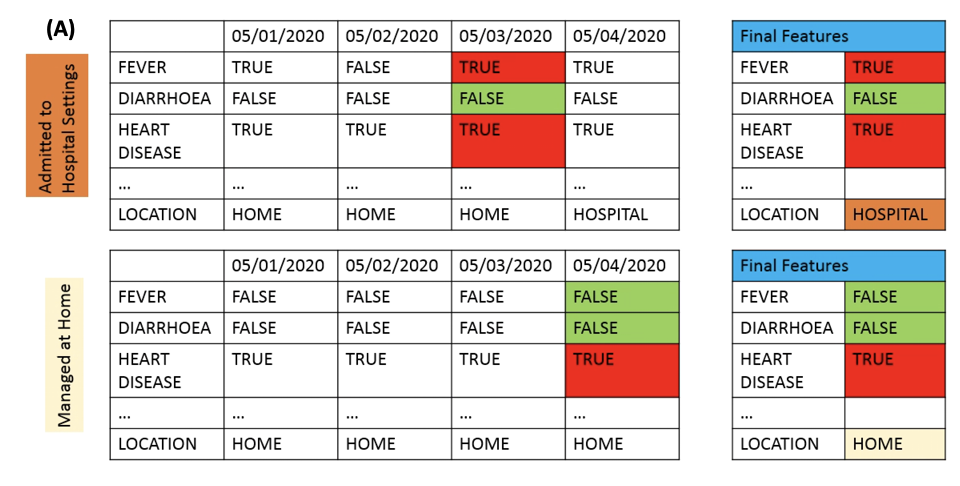
**


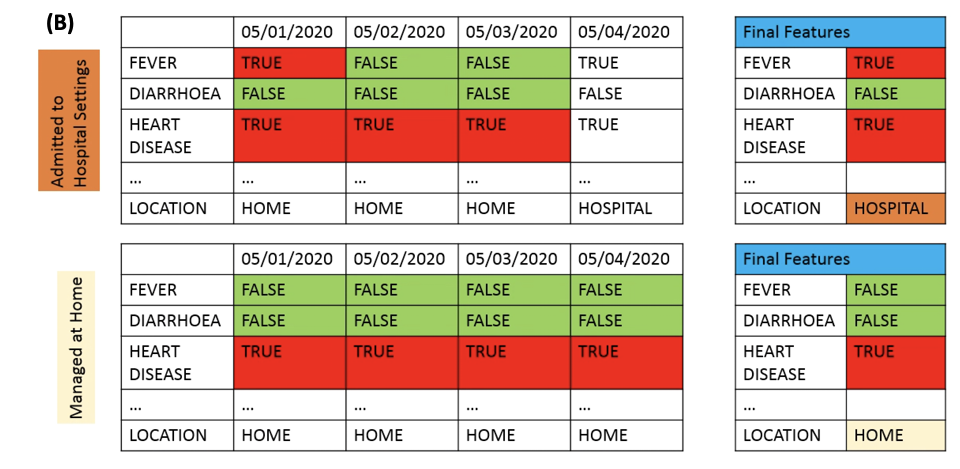


**Figure S2. Usage of the features.** On the top panel, for users who were seeking treatment, we used the time point right before a user indicated he/she is in a hospital setting and the features at that time point for analysis. For users who were always at home, we used the last time point and the features at that time point for analysis on the bottom panel, for users who were admitted to a hospital setting, if a user indicated that he/she had a feature in any of his/her entire entries before the day of being admitted in a hospital setting, we labeled that feature as positive for that user. For users who were always at home, if he/she had a feature for his/her entire entry log, we labeled that feature as positive for that user. Such methods only apply to symptoms since they can change every day and not to comorbidities, pre-existing medication use, or demographics.

**
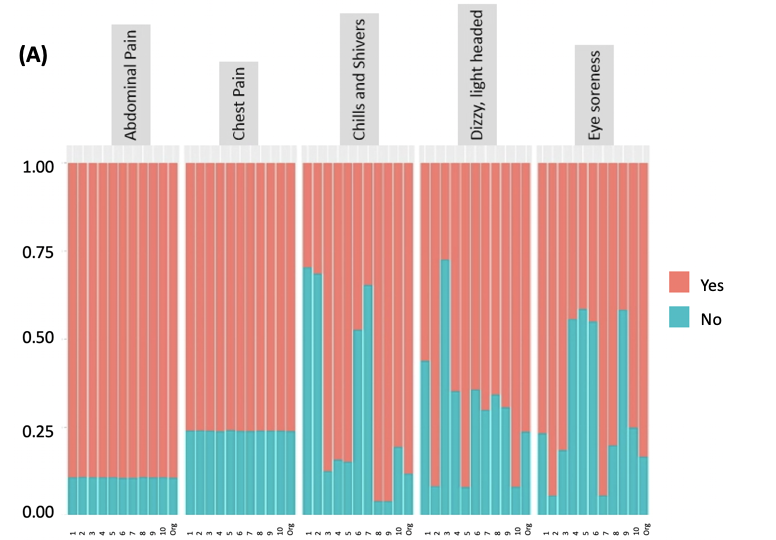
**


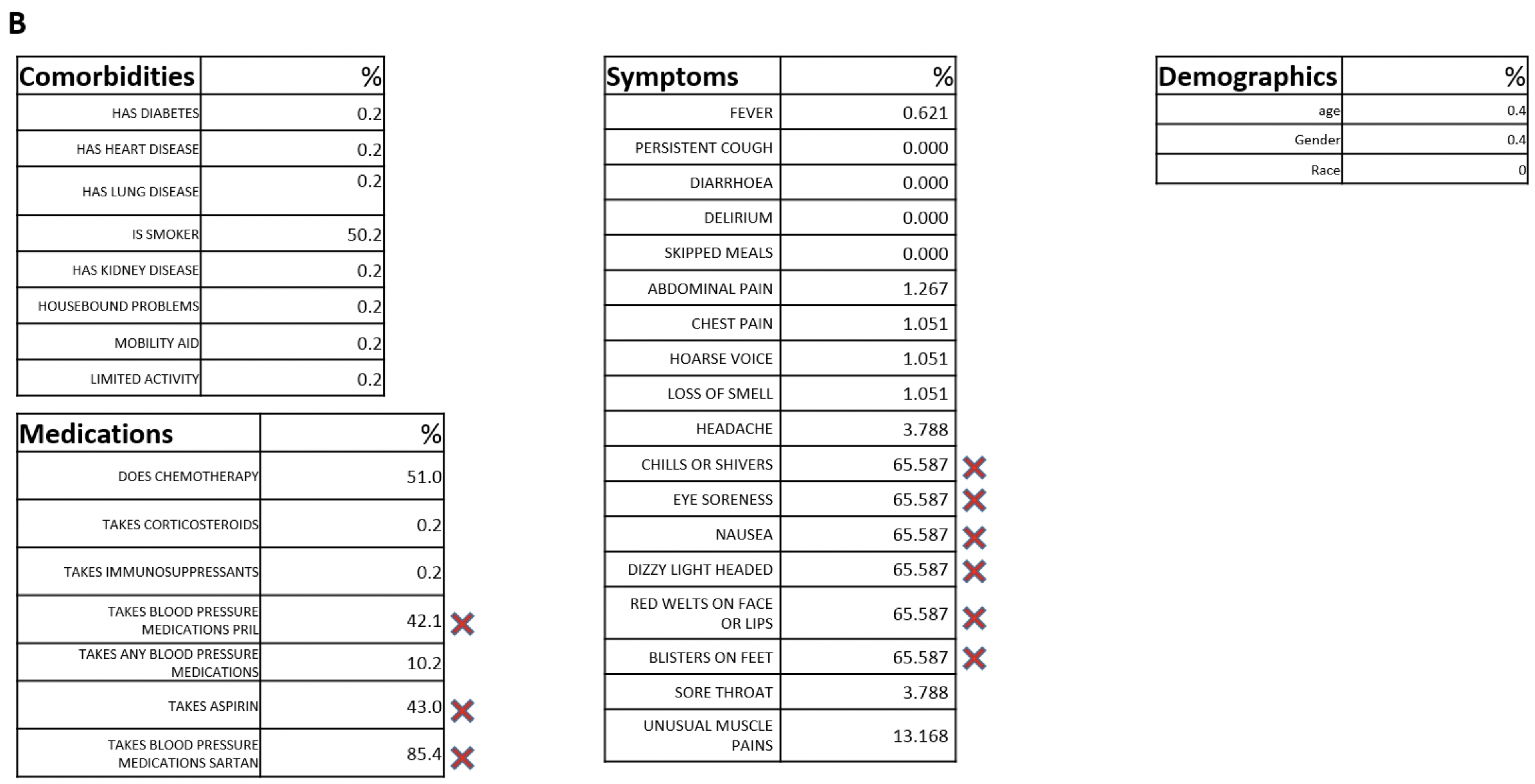


**Figure S3: Multiple Imputation of missing values**. An example of the original distribution of features and imputed distribution of those features is shown in the table. The last column of each feature group labeled ‘org’ is the original distribution without imputation. Labels 1-10 are the ten different distributions after multiple imputations of the missing values. Some features, ‘Abdominal pain’ and ‘Chest pain’ in this example are able to retain the original distribution after multiple imputations. Other features had a wide range of distributions that were wildly different from the original, indicating the multiple imputations for these features were not suitable. Those features were removed from the original data set. The features removed are labeled with a red cross in the figure on top**.** The percentage missing is shown for each feature.

**
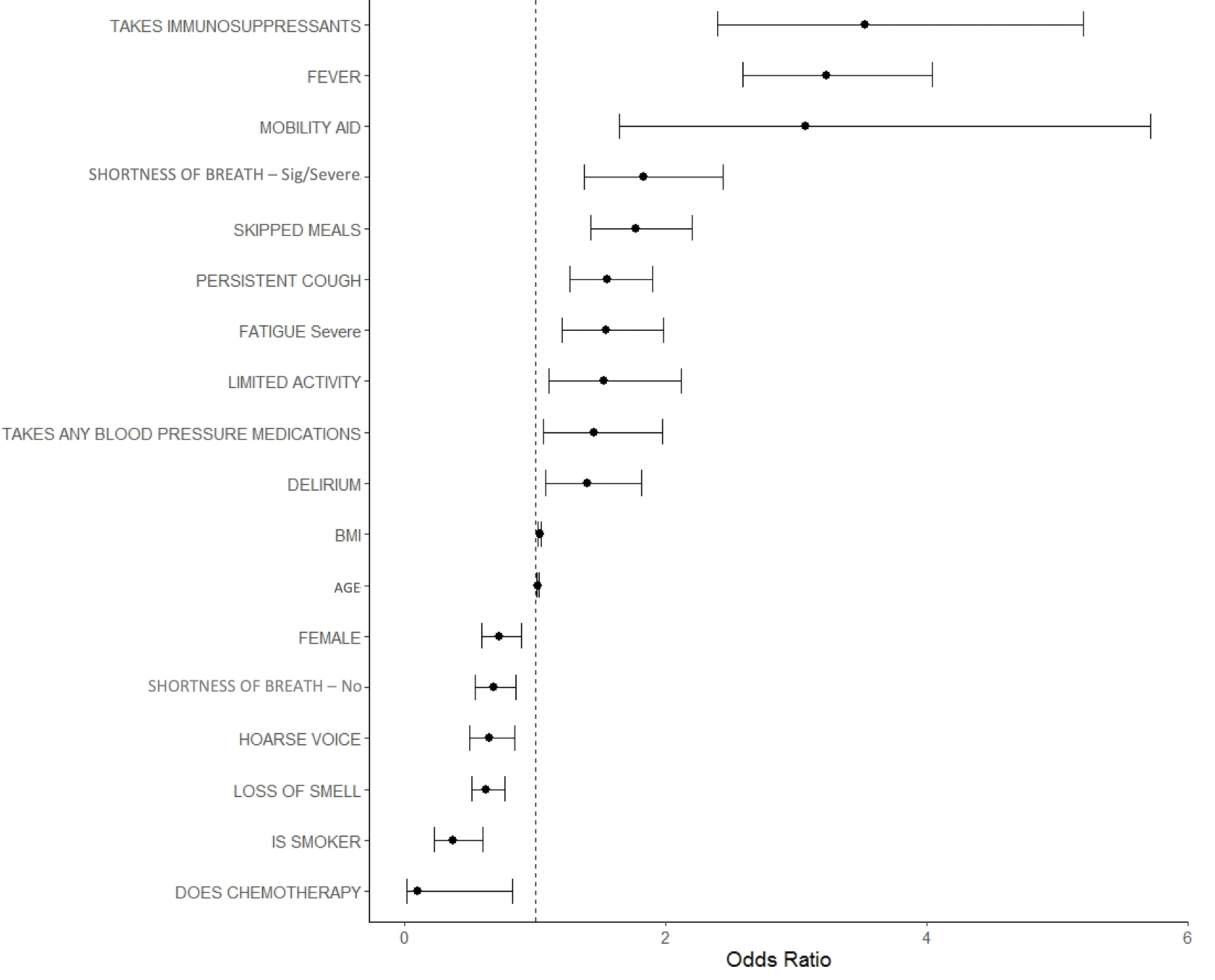
**

**Figure S4: Estimated Odds Ratios for each potential risk factor from a logistic regression model.** Error bars represent 95% confidence interval for the odds ratio. All odds ratios are adjusted for all other factors listed. Only Significant features are shown.

**
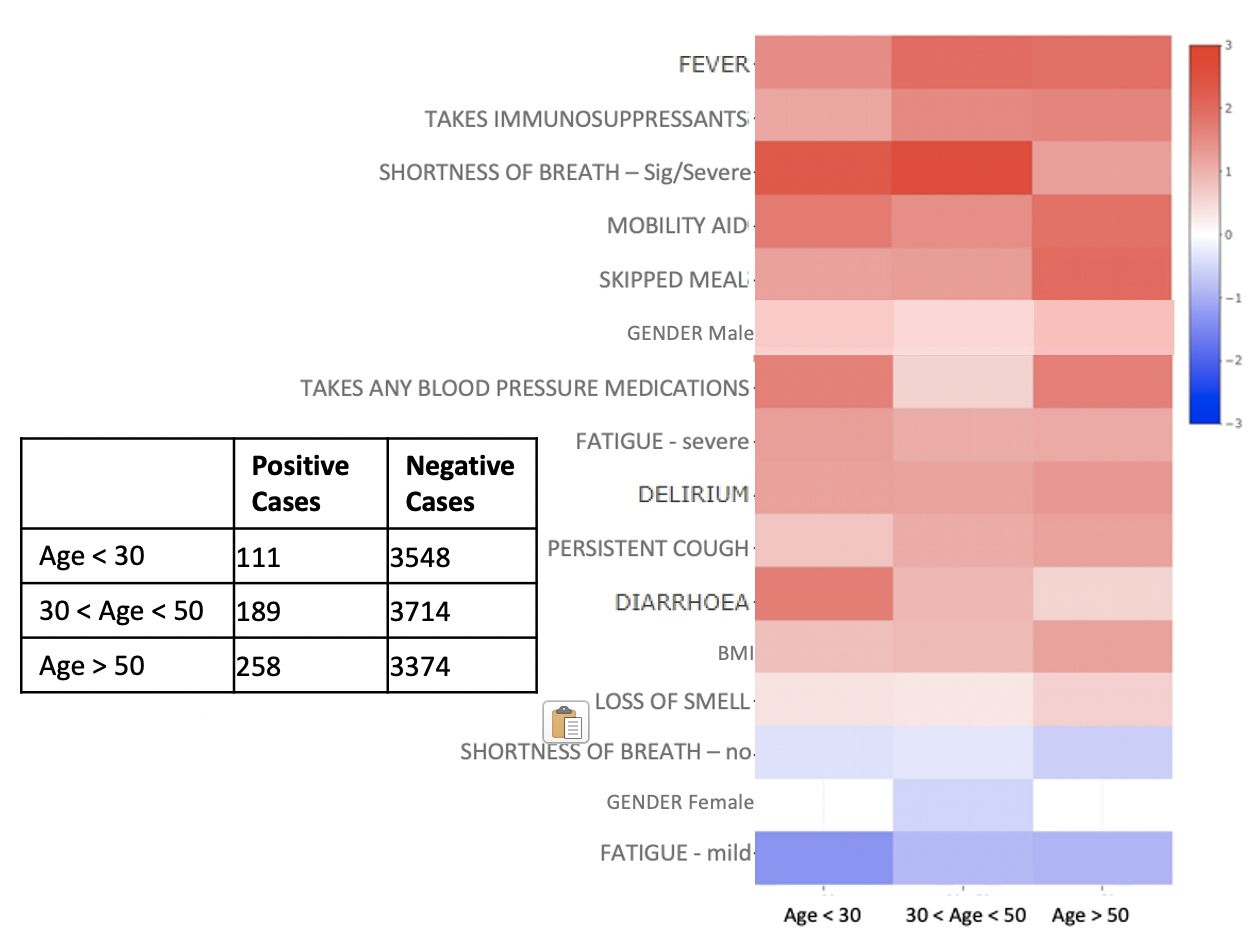
**

**Figure S5. Univariate Logistic Regression of young, middle age, and old age groups.** All the users with COVID-19 were divided into three groups of young, middle age, and old age groups. The number of positive cases (seeking treatment) and the number of negative cases (stayed home) are shown. The outcome of whether a user was seeking treatment was regressed onto each of the features selected by the Elastic Net Regression. The coefficients for each feature for each age group is plotted. Only significant ones are colored. The three groups have similar patterns of expression in the features selected.

**
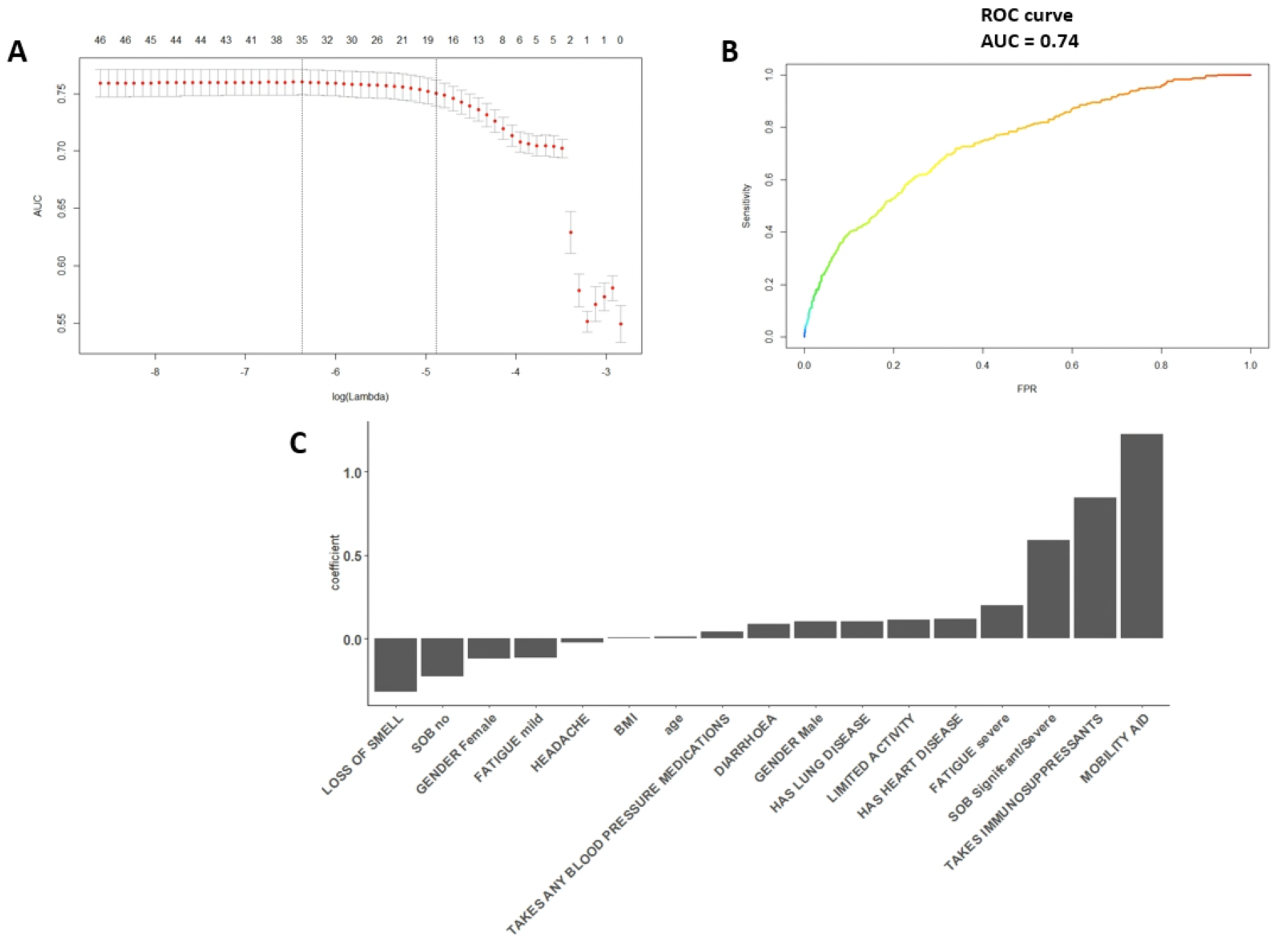
**

**Figure S6: Results of Elastic Net Regression using scenario 2**. Scenario 2 where for each feature, if a user indicated he/she had that feature in any of his/her entire entries, we labeled that feature as positive for that user. The performance in terms of cross-validation area under the Receiver Operating Curve (AUC) for validated Elastic Net Regression on the training set across different values of lambda. The AUC of the trained Elastic Net model applied on a holdout test data set. The most important features selected by the Elastic Net model. Negative coefficients indicate a negative association with outcome and vice versa. Features selected are similar to scenario 1. Predictive performances are also comparable.

**
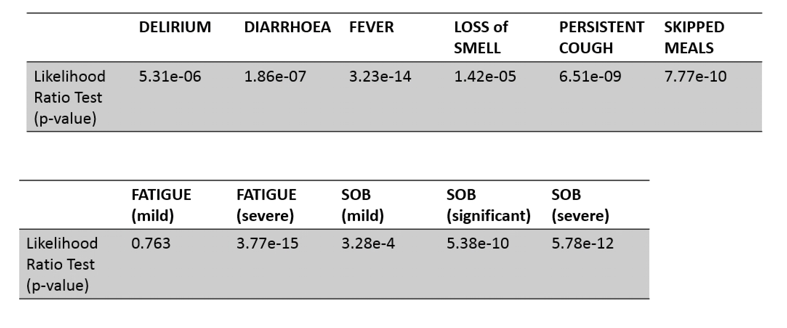
**

**Figure S7. Likelihood ratio test between positive and negative groups.** A 20 days window was examined for positive and negative cases. For each day, the frequency of users having the feature for the positive and negative groups is plotted. Linear regression where the frequency is regressed on the days before the last day. Slope and intercepts were obtained and the likelihood ratio test was used to evaluate whether the slopes were statistically different. P-value < 0.05 indicates the positive and negative groups have statistically different slopes.


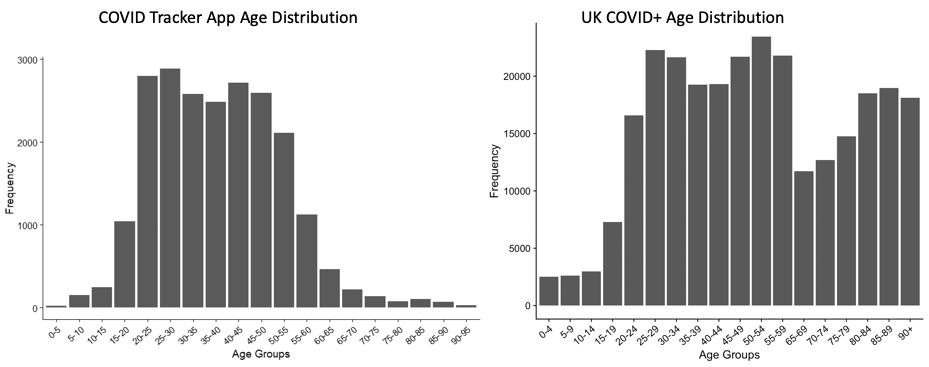


**Figure S8.** **Age distribution comparison between population in COVID Tracker App vs. population of UK tested COVID positive.** The age distribution shown on the left is for the population in the COVID Tracker App of those tested positive for COVID-19. The distribution is unimodal with an average of 44.3. The age distribution shown on the right is for the general UK population tested positive for COVID-19 between the period of January 2020 - September 2020. The average age is older at 57.6 with a large proportion of older population. This difference in age distribution could contribute to the small age coefficients of our model compared to current literature.
